# Calibration-Curve-Locking Database for Semi-Quantitative Metabolomics by Gas Chromatography/Mass Spectrometry

**DOI:** 10.3390/metabo11040207

**Published:** 2021-03-30

**Authors:** Kosuke Hata, Yuki Soma, Toshiyuki Yamashita, Masatomo Takahashi, Kuniyo Sugitate, Takeshi Serino, Hiromi Miyagawa, Kenichi Suzuki, Kayoko Yamada, Takatomo Kawamukai, Teruhisa Shiota, Yoshihiro Izumi, Takeshi Bamba

**Affiliations:** 1 Division of Metabolomics, Research Center for Transomics Medicine, Medical Institute of Bioregulation, Kyushu University, 3-1-1 Maidashi, Higashi-ku, Fukuoka 812-8582, Japan; k-hata@bioreg.kyushu-u.ac.jp (K.H.); y-soma@bioreg.kyushu-u.ac.jp (Y.S.); toshiyuki_yamashita@bioreg.kyushu-u.ac.jp (T.Y.); m-takahashi@bioreg.kyushu-u.ac.jp (M.T.); 2 Agilent Technologies Japan Ltd., 9-1 Takakuramachi, Hachioji-shi, Tokyo 192-8510, Japan; kuniyo_sugitate@agilent.com (K.S.); takeshi_serino@agilent.com (T.S.); 3 GL Sciences Inc., 6-22-1 Nishi-Shinjuku, Shinjuku-ku, Tokyo 192-8510, Japan; h.miyagawa@gls.co.jp (H.M.); suzuki@gls.co.jp (K.S.); 4 AMR Inc., 2-13-18 Nakane, Meguro-ku, Tokyo 192-8510, Japan; k-yamada@amr-inc.co.jp (K.Y.); t-kawamukai@amr-inc.co.jp (T.K.); t-shiota@amr-inc.co.jp (T.S.)

**Keywords:** calibration-curve-locking-database, quantification, metabolomics, GC/MS, Standard Reference Material (SRM) 1950, data integration

## Abstract

Calibration-Curve-Locking Databases (CCLDs) have been constructed for automatic compound search and semi-quantitative screening by gas chromatography/mass spectrometry (GC/MS) in several fields. CCLD felicitates the semi-quantification of target compounds without calibration curve preparation because it contains the retention time (RT), calibration curves, and electron ionization (EI) mass spectra, which are obtained under stable apparatus conditions. Despite its usefulness, there is no CCLD for metabolomics. Herein, we developed a novel CCLD and semi-quantification framework for GC/MS-based metabolomics. All analytes were subjected to GC/MS after derivatization under stable apparatus conditions using (1) target tuning, (2) RT locking technique, and (3) automatic derivatization and injection by a robotic platform. The RTs and EI mass spectra were obtained from an existing authorized database. A quantifier ion and one or two qualifier ions were selected for each target metabolite. The calibration curves were obtained as plots of the peak area ratio of the target compounds to an internal standard versus the target compound concentration. These data were registered in a database as a novel CCLD. We examined the applicability of CCLD for analyzing human plasma, resulting in time-saving and labor-saving semi-qualitative screening without the need for standard substances.

## 1. Introduction

Recently, the demand for quantitative metabolomics to derive metabolite concentrations has increased with the expansion of research fields that require a data comparison across measurement batches, methods, and facilities (e.g., cohort studies, international collaborative research, pharmacokinetic analysis, and trans-omics research) [1,2,3]. Chromatography coupled with mass spectrometry (MS) (e.g., gas chromatography/mass spectrometry, GC/MS, liquid chromatography/mass spectrometry, LC/MS, and capillary electrophoresis/mass spectrometry, CE/MS) are the most commonly used techniques in metabolomics because they allow the identification of a wide range of molecular species [4]. However, it is not easy to guarantee quantitative performance with mass spectrometry-based metabolomics because procedures for experiments and data processing are highly complex and error-prone [5]. In particular, it is necessary to obtain calibration curves for numerous target metabolites for each experiment because the detection sensitivity for each quantifier ion in mass spectrometry generally fluctuates day by day. Hence, obtaining metabolite concentrations from mass spectrometry-based metabolomics is labor-intensive.

GC/MS is utilized as a primary method for metabolomics because of its high sensitivity, peak capacity, and repeatability, especially for low–molecular-weight metabolites [6]. In addition, its compound identification capabilities are superior to those of other techniques owing to the reproducible fragmentation patterns by the electron ionization (EI) mass spectra, in which the electron acceleration energy is unified at 70 eV. In particular, the retention time (RT) of peaks obtained by GC analysis can be constant among the analysis batches by employing the retention time locking (RTL) method. Therefore, the repeatability of the GC separation can also be improved. Taking advantage of these features of GC/MS, public databases have been constructed for GC/MS-based metabolomics with accurate metabolite identification [7,8,9]. The most versatile and large-scale database for GC/MS-based metabolomics has been reported by Kind et al. as Fiehn Library, in which more than 1000 compounds have been registered [7]. Furthermore, several research groups have constructed and updated GC/MS libraries for target/non-target metabolome analyses [8,9,10].

In addition to these advantages, a quantification methodology based on GC/MS was developed by constructing a calibration curve locking database (CCLD) in the fields of environmental analysis, pesticide analysis, and forensics [11,12,13]. CCLD includes the RT, EI mass spectrum, and calibration curve for each target compound. Under target tuning methods of MS such as a decafluorotriphenylphosphine (DFTPP) tuning in the method provided by the United Sates Environment Protection Agency (US EPA method 625) (https://www.epa.gov/sites/production/files/2015-10/documents/method_625_1984.pdf, latest accessed on 1 February 2021), the calibration curves based on the relative peak area (RPA) are constant even if the absolute sensitivity of mass spectrometry fluctuates. Therefore, CCLD enables the quantification of metabolites without the day-to-day preparation of calibration curves by measuring standard substances.

**Figure 1:**
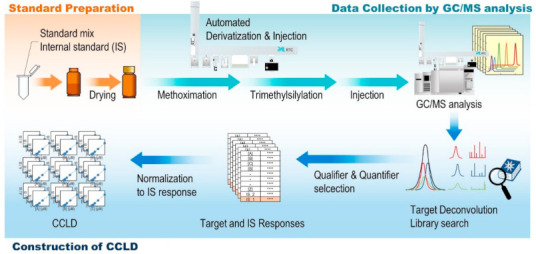
Conceptual diagram showing workflow for construction of a novel calibration curve locking database (CCLD) for metabolome analysis. The novel CCLD was constructed for semi-quantitative screening by gas chromatography/mass spectrometry (GC/MS). Our in-house CCLD contains the retention time (RT), calibration curve, and electron ionization (EI) mass spectrum for automatic compound search and semi-quantification of target compounds. To achieve repeatable quantification by using CCLD, automated batch and in-time sample derivatization and sample loading protocol by a PAL RTC system was employed for the construction of the CCLD.

In this study, we attempted to apply these CCLD concepts to GC/MS-based metabolomic analysis. Because the quantitative performance and identification accuracy of CCLD can be secured as long as the apparatus conditions remain constant, development of methods for apparatus conditioning were necessary. Most of the targets in previous CCLDs are compounds that do not require derivatization, such as organic compounds, agricultural chemicals, and other drugs [11,12,13]. On the other hand, many metabolites are non-volatile and require derivatization. To ensure the quantitative performance of GC/MS analysis with CCLD, it is necessary to stably reproduce the derivatization reaction for each measurement [14,15,16]. To this end, automatic derivatization systems have been developed using an auto-sampler and robotic platforms [17,18,19,20]. We employed a robotic platform system (i.e., a multifunction automatic sampler, PAL RTC system) for the automation of sequential sample manipulation including two-step derivatization and injection to GC/MS (Figure 1). After optimization of the automated sequential two-step derivatization for oximation and trimethylsilylation, we collected calibration curves of 52 metabolites in central carbon metabolism under DFTPP tuning conditions. After verifying the stability of the calibration curves over several days, calibration curves were registered in the EI spectrum database based on the Fiehn Library, resulting in a novel CCLD for metabolomics. A novel CCLD was validated by its quantity and repeatability for quantification of reference biological samples, Standard Reference Material (SRM) 1950, provided by the National Institute of Standards and Technology (NIST).

## 2. Results and Discussion

### 2.1. Verification of Stability of Relative Sensitivity of Mass Spectrometry

**Figure 2:**
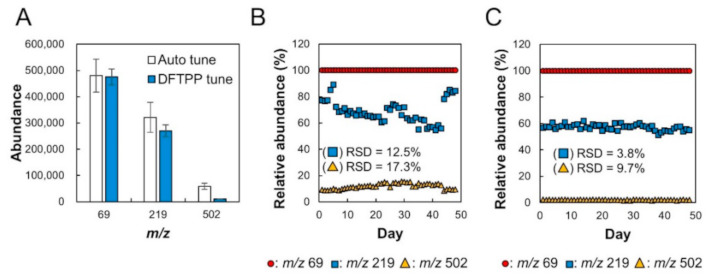
Differences in target ion count due to the MS tuning condition between auto tuning and decafluorotriphenylphosphine (DFTPP) tuning. (**A**) Average absolute abundance (ion count per second) over 48 days at *m*/*z* = 69, 219, and 502. Open bars indicate the results obtained using the auto tuning method, and black bars indicate that obtained using the DFTPP tuning method. Error bars represent the standard deviation (*n* = 3). (**B**,**C**) Tuning logs of the auto tuning and DFTPP tuning, respectively, represented by the time course of relative abundance.

For the construction of the CCLD, the relative sensitivity among each *m*/*z* of mass spectrometry should be kept constant by DFTPP tuning [10]. The actual sensitivity fluctuation was monitored by auto tuning and DFTPP tuning for 48 days (Figure 2). The absolute sensitivity for *m*/*z* = 69, 219, and 502 under the auto tuning method fluctuated with relative standard deviations (RSDs) of 13.1%, 18.0%, and 20.4%, respectively. Under DFTPP tuning, the fluctuations in the abundance of *m*/*z* = 69, 219, and 502 were 6.5%, 8.2%, and 11.0%, respectively. The auto tuning algorithm sets the parameters to maximize the mid-range and high-end sensitivity (i.e., high abundances of ions 219 and 502). On the other hand, that of the DFTPP tuning sets the target relative abundances of *m*/*z* = 69, 219, and 502 to 100%, 55%, and 2%, respectively. Under auto tuning, the relative abundance ratio of *m*/*z* = 219 and 502 normalized by *m*/*z* = 69 fluctuated with RSDs = 12.5% and 17.3%, respectively, according to the fluctuation of absolute sensitivity for each *m*/*z* (Figure 2B). In the case of DFTPP tuning, the fluctuation of the relative abundance ratio between each *m*/*z* was 3.8% and 9.7% (Figure 2C). These results showed that the RPA for the calibration curve of each target metabolite remained constant among different analytical batches by DFTPP tuning.

### 2.2. Optimization of Automatic Derivatization Condition Using PAL RTC

For the metabolome analysis based on GC/MS, a two-step sequential derivatization reaction was employed, which combines oximation and trimethylsilylation. The method provided by the Fiehn group enabled efficient derivatization by decreasing the amount of oxime reagent and increasing the amount of silylating reagent [6]. We employed Fiehn’s method for the automation of the two-step sequential derivatization using a robotic platform PAL system, according to our previous report [21]. For the construction of a CCLD ensuring high repeatability and quantitative performance, it is necessary to improve the sensitivity of GC/MS because the absolute sensitivity under the DFTPP tuning tended to be lower than under auto tuning, as shown in Figure 2. The reaction temperature and reagent amount for the two-step sequential derivatization reaction were modified to improve the sensitivity of the GC/MS-based metabolomic analysis. To evaluate the improvement in sensitivity by the modification of the derivatization method, standard substance mixtures (SSMs) of 52 metabolites (Table 1) were analyzed, and the relative peak area (RPA), which is the normalized peak area of the quantifier of target metabolites by that of the internal standard (IS), were compared for each method.

For comparison of the method, SSM and IS_1_ (d_10_-phenanthrene) were prepared so that their theoretical final concentration after the derivatization process was set to constant, [SSM] = 200 μmol/L and [IS_1_] = 53.1 μmol/L (10 μg/L) (Table S1). To unify the agitators for automatic derivatization using the PAL system, the same temperature conditions were applied to both the oximation and silylation reaction. We compared RPA of each metabolite among method A (30 °C), B (37 °C), and C (50 °C) using the PAL system. As a control method, we employed a manual derivatization method provided by the Fiehn group (oximation at 30 °C and silylation at 37 °C), as shown in Table S1. All detected TMS derivatives of the target metabolites are listed in Table S2. One derivative was chosen for each metabolite as a quantification target based on sensitivity and repeatability (Table 1). No significant differences in RPA values were observed between the control method and method A (30 °C) (Table S3). With methods B (37 °C) and C (50 °C), the RPA values of α-ketoglutarate and cysteine increased. The RPA values of fumaric acid and glutamic acid decreased, while those of pyroglutamic acid increased at 50 °C. These results indicate that increasing the temperature increased the derivatization efficiency for α-ketoglutarate and cysteine, while a high temperature caused the conversion of glutamic acid to pyroglutamic acid. Therefore, we employed 37 °C for the derivatization reaction. Next, to achieve higher sensitivity by reducing the sample dilution with a derivatization reagent, the reagent volumes of oximation and silylation were reduced (methods D and E in Table S1). In the case where the total volume of reagent for oximation and silylation was reduced by 50% (method D), almost all target metabolites were detected with higher RPA values compared with those of the control method (Table S3). A further 25% reduction in the volume of the silylation reagent (method E) was achieved with higher RPA values than that obtained using the control method for almost all target metabolites (Table S3).

**Figure 3:**
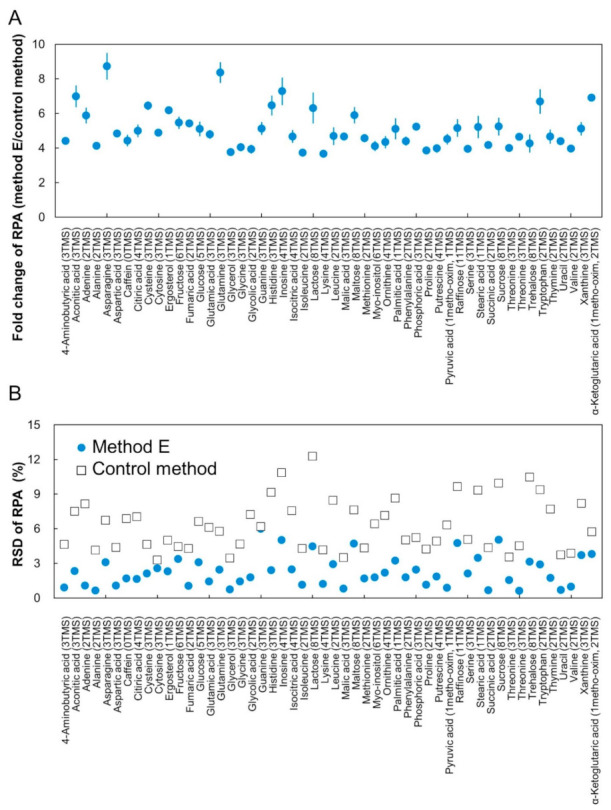
Improvement in sensitivity by the optimization of derivatization reaction. Each RPA with method E was dividing by that with control method, which was defined as the fold change of RPA (**A**). Repeatability of RPA for each metabolite is shown in (**B**). Error bars represent the standard deviation (*n* = 3).

Considering these results, method E was chosen as the optimized derivatization method for the novel CCLD construction. Finally, we compared the GC/MS analysis results of the same analyte (25 μL SSM containing 200 μmol/L for each metabolite) obtained using method E and the control method. Figure 3A shows the fold changes of RPA (the value obtained by dividing the RPA with method E by the RPA with the control method) for all target metabolites. The improvement in sensitivity using method E was over four-fold among all 52 metabolites (Figure 3A). This improvement in sensitivity was mainly due to the concentration effect by a reducing reagent volume, whereas improvement in the derivatization efficiency by changing the temperature also enhanced the sensitivity for some metabolites. The repeatability of the RPA value of each metabolite also improved with increasing sensitivity (Figure 3B).

### 2.3. Construction of CCLD

To prepare calibration curves for 52 metabolites for CCLD, 0, 5, 50, 100, and 200 μM of SSMs were analyzed under DFTPP tuning. Three additional concentrations (500, 1000, and 1500 μM) were analyzed for glucose because the standard reference sample SRM 1950 contained glucose at this concentration range. Each calibration curve was prepared as a linear calibration by least square regression using the RPA normalized by IS_1_ (d_10_-phenanthrene) (Figure S1). All concentrations were analyzed in triplicate for three different days, resulting in nine calibration curve sets and one averaged calibration curve set (Table 1). Intra-day and day-to-day variations in the slope of each calibration curve were small for all metabolites (Table S4). These results clearly demonstrated that DFTPP tuning facilitated the repeatable analysis of the same analytes among different analytical batches. The limits of quantification (LOQ) was determined by analysis of serially diluted SSMs. The LOQ was defined as the lowest analyte concentration at which the signal-to-noise ratio (*S*/*N* ratio) of over 10 was detected. The LOQs were 50 μM for the following metabolites: ergosterol, glycine, glycolic acid, pyruvic acid, and α-ketoglutaric acid. The LOQs were 5 μM for all other metabolites (Table S4). The average calibration curve set, mass spectra, and other parameters listed in Table 1 (RT, quantifier, and qualifiers) were registered to a quantification method using MassHunter software, resulting in the novel CCLD.

### 2.4. Method Validation by Quantification of Human Plasma Sample

Finally, we performed method validation of CCLD-based GC/MS quantification using a reference sample (50 μL) of human plasma NIST SRM 1950. To determine the appropriate sample pretreatment, different volumes of plasma extract (upper phase 50, 100, and 150 μL, see Materials and Methods for details) were analyzed, and the range where the RPA value was linear was determined (Table S5). Since linearity was lost with fructose and succinic acid in the case of 150 μL of a sample volume (Figure S2). The amount of plasma extract was set to 100 μL afterwards. To validate the constructed CCLD, an addition-recovery test was performed using SRM 1950. A mixture of 25 μL of SSM (50 μM) and 100 μL of plasma sample extract was prepared in a vial and analyzed by GC/MS using CCLD. As a result, a reasonable recovery rate was achieved for all metabolites (58–133%) (Table 2).

To evaluate the quantification by a novel CCLD for metabolomics, reference human plasma sample SRM 1950 was analyzed, and the quantification results were compared with the metabolite concentrations provided by NIST. We analyzed SRM 1950 three times per day, and the analysis was performed for three days. As a result, 28 metabolites in CCLD were detected and identified (Table 3). The quantified concertation of each identified metabolite by the novel CCLD was close to the literature values provided by NIST (https://www-s.nist.gov/srmors/certificates/1950.pdf (accessed on 1 February 2021)).

Although we confirmed that adipic acid was not detected from SRM1950, several studies mentioned that adipic acid can be included in human blood as an exogenous food additive [22,23,24]. In order to improve the quantitative performance of CCLD, it is helpful to use commercially available stable isotopope-labeled adipic acid as an alternative internal standard (e.g., ^13^C_2_-adipic acid). To expand the variation of CCLD for different types of biological sample (e.g., foods, cells, tissue, urine, and feces), there is room to select appropriate internal standards for each sample type and target metabolites.

From these results, it was proven that CCLD could be used for quantitative metabolome analysis over different batches with high repeatability (except for metabolites that have been recognized as difficult to quantify). In the analysis of SRM 1950, 28 metabolites were detected in addition to the 52 target metabolites registered in the CCLD. Since the CCLD is based on non-target GC/MS analysis, if there are interesting metabolites, it is possible to quantify them even in past data measured in different batches by updating the CCLD with the addition of new calibration curves. By analyzing various biological samples with CCLD and expanding the library, it is expected that the usefulness of metabolome data would be synergistically increased.

## 3. Materials and Methods

### 3.1. Material and Reagents

Methoxyamine hydrochloride (quality level: MQ 200) was purchased from Merck (Darmstadt, Germany). Pyridine (extra dry) (99.5+% evaluated by capillary GC), acetone (Dioxins Analysis Grade, purity 99.8%), and d_10_-phenanthrene (Environment Analysis Grade, purity 99.5%) were obtained from Wako Chemicals Ltd. (Osaka, Japan). The metabolite mixture kit for GC/MS-based metabolomics and *N*-methyl-*N*-(trimethylsilyl trifluoroacetamide) (MSTFA) were purchased from GL Sciences Inc. (Tokyo, Japan). Acetonitrile (LC-MS grade) and methanol (LC-MS grade) were purchased from Kanto Chemical (Tokyo, Japan). Standard Reference Material (SRM) 1950 “Metabolites in Human Plasma” was obtained from the National Institute of Standards and Technology (NIST, Gaithersburg, MD). Adipic acid (purity 99.5%), chloroform (purity 99.0%), and methanol (purity 99.8%) were obtained from Nacalai Tesque Inc. (Kyoto, Japan).

### 3.2. Preparation of Standard Mixtures

To prepare the calibration curves of target metabolites, we used the metabolite mixture kit as the SSM, which contains 52 metabolites (200 μmol/L each) related to central carbon metabolism (Table 1). SSM was serially diluted by water to obtain an appropriate range of concentration, and 100 μL each was transferred to a clean 300 μL fixed-insert vial. They were dried for 2 h using a centrifugal concentrator (VC-36S, TAITEC Co., Saitama, Japan). During this drying step, the inner wall of the vial insert was washed three times with methanol to wash down the compounds that stick to the insert wall into the reaction solution. To gradually reduce the liquid level in the insert, we decreased the washing methanol volume step-by-step to 50 μL at 20 min, 20 μL at 40 min, and 10 μL at 55 min. When the applied volume of SSM in each method was changed to optimize the derivatization conditions, we adjusted the SSM concentration to ensure that the on-column concentration of each metabolite was the same.

### 3.3. Extraction of Metabolites from the Plasma Sample

Each 50 μL of the plasma sample was collected in a clean 2 mL microtube. It was diluted with 950 µL of extraction solution containing 546 μL of methanol, 222 μL of chloroform, 172 μL of water, and 10 μL of adipic acid (1 mmol/L in stock) as IS_2_ (Figure S4). After vortex mixing, the mixture was centrifuged at 16,000× *g* at 4 °C for 5 min, and then the upper phase (700 μL) was transferred to a clean 2 mL microtube. After the addition of 155 μL of water and 235 μL of chloroform, the mixture was vortexed and centrifuged at 16,000× *g* at 4 °C for 3 min. The upper phase (100 μL) was transferred to a clean 300 μL fixed-insert vial and evaporated to dryness, same with the SSM drying step described above. The dried plasma extract was subjected to automated derivatization and GC/MS analysis, as described in the next section.

### 3.4. Automated Derivatization and GC/MS Analysis

The method for sequential automatic derivatization and injection to GC/MS analysis was constructed using the robotic platform PAL RTC and PAL Sample Control software (CTC Analytics AG, Zwingen, Switzerland). The configuration of the PAL RTC system is shown in Figure S3. A brief summary of the automatic sequential derivatization and injection procedure is presented in Figure S4. The vials containing the derivatization reagents and the sample were placed on a vial tray on the tray holder of the PAL RTC system (Figure S3B). Oximation reagent (pyridine containing 40 mg/mL of methoxyamine hydrochloride and d_10_-phenanthrene as IS_1_) and MSTFA were placed into clean vials on tray 2 on slot 2 of the tray holder. Concentration of d_10_-phenanthrene in the oximation reagent was changed so that the theoretical final concentration after the derivatization is 53.1 mmol/L (10 μg/L) when the total volume of the silylation reagent and the oximation reagent were changed.

The vial with dried plasma extracts was capped with a metal cap (GL Sciences Inc., Tokyo, Japan) for the PAL system and placed on tray 1 on slot 1 of the tray holder. To start the derivatization process, a vial with the sample was moved to the agitator, and 5 µL of the oximation reagent was added to dissolve the dried sample and mixed. The sample was mixed for 90 min at 750 rpm and 37 °C in an agitator. The second reagent (20 µL), MSTFA, was then added to the sample and mixed for 30 min at 750 rpm and 37 °C. After 2 h of the completion of derivatization, the vial was moved back to tray 1.

Agilent GC 7890A coupled to a 5975C inert MSD (Agilent Technologies, Santa Clara, CA, USA) was used for metabolomic analysis. For this purpose, 1 µL of the sample was injected using a 10 μL syringe of PAL RTC. GC analysis was performed on a DB5-MS (i.d.: 30 m × 0.25 mm, film thickness: 0.25 μm) capillary column (Agilent Technologies). Prior to sample analysis, the RT was locked at 16.727 min using d_27_-trimethylsilylated myristic acid [10]. The samples were injected in a split mode (1:10) with the injection port held at 250 °C. The initial oven temperature was maintained at 60 °C for 1 min and then ramped at 10 °C/min to 325 °C and held for 10 min. The MSD transfer line was held at 290 °C, the ion source was held at 250 °C, and the quadrupole was held at 150 °C. EI mass spectra were generated at an ionization energy of 70 eV. The GC/MS data were acquired at 37.5 min with 5.9 min of solvent delay at a normal scan rate (781 u/s) in the mass range of *m*/*z* 50–650. DFTPP tuning was performed to obtain uniform mass spectra using a DTFPP tune file provided by Agilent Technologies, which allows adjusting MS parameters to meet relative abundance criteria defined by EPA methods 625 (https://www.epa.gov/sites/production/files/2015-10/documents/method_625_1984.pdf, accessed on 1 February 2021).

### 3.5. Data Analysis

Data analysis was performed using Mass Hunter B.07.01 (Agilent Technologies). All quantifier ions for the target metabolites and ISs are listed in Table S2. After peak detection with deconvolution, the target compound peaks were identified by a library search. The peak area of each quantifier ion was then determined. The peak area of d_10_-phenanthrene (*m*/*z* 188) as IS_1_ was used to normalize all the other peak areas. The extraction efficiency of the plasma sample in the sample preparation was corrected based on the peak area of adipic acid (*m*/*z* 111) as IS_2_.

### 3.6. Preparation of Calibration Curves

SSMs were prepared for the calibration curve construction so that the theoretical concentration after the derivatization was set to 0, 5, 50, 100, and 200 μmol/L. For glucose, three additional concentrations of 500, 1000, and 1500 μmol/L were analyzed. As the IS_1_, d_10-_phenanthrene was prepared at a concentration of 25 μg/mL in the oximation reagent (pyridine containing 40 mg/mL methoxyamine hydrochloride) to obtain a theoretical final concentration of 53.1 μmol/L (10 μg/L). Each calibration curve was prepared for linear calibration by least square regression using the plot of RPA normalized by IS_1_.

## 4. Conclusions

We constructed a novel CCLD for quantitative metabolomics, including EI mass spectrum, RT, quantifier ion, and calibration curves, for 52 metabolites in central carbon metabolism. The derivatization reaction was automated using a robotic platform to ensure high repeatability in the GC/MS analysis with CCLD. We improved the sensitivity for each metabolite over four times by optimizing the conditions for the derivatization reaction to ensure quantitative performance. Using SRM 1950 from NIST as a reference biological sample, we demonstrated that quantification of metabolites using CCLD showed high repeatability in different batches. Quantification of SRM 1950 by CCLD was similar to that provided by NIST. Since CCLD has extensibility and MS scan data measured under target tuning can be reused, it is expected that the more biological samples analyzed, the greater the usefulness of the accumulated data. For further expansion of application of CCLD to various types of biological samples (e.g., foods, cells, tissue, urine, and feces), there is room to investigate matrix effects and selection of appropriate internal standards for each sample type and target metabolites.

## Figures and Tables

**Table 1:** Data collection for CCLD construction.

ID	Metabolite	RT (min)	Quantification Ion	Qualifier Ion 1	Qualifier Ion 2	Slope	Intercept	*R* ^2^
		Mean ± SD (*n* = 9)	(*m*/*z*)	(*m*/*z*)	(*m*/*z*)	(*n* = 9)	(*n* = 9)	
M001	4-Aminobutyric acid (3TMS)	13.31 ± 0.01	304	174	147	0.00428	−0.01677	0.997946326
M002	Aconitic acid (3TMS)	15.81 ± 0.01	147	375	229	0.00939	−0.10858	0.971925122
M003	Adenine (2TMS)	17.12 ± 0.01	264	279	192	0.0145	−0.1298	0.985093709
M004	Alanine (2TMS)	7.43 ± 0.01	116	190	147	0.0181	−0.0537	0.998107977
M005	Asparagine (3TMS)	14.93 ± 0.01	116	231	132	0.00296	−0.03480	0.969363443
M006	Aspartic acid (3TMS)	13.16 ± 0.01	232	218	100	0.0148	−0.1094	0.990087236
M007	Caffeine (0TMS)	17.01 ± 0.01	194	109	67	0.00655	−0.04092	0.992869316
M008	Citric acid (4TMS)	16.55 ± 0.01	273	465	347	0.0186	−0.1197	0.993577885
M009	Cysteine (3TMS)	13.6 ± 0.01	218	220	100	0.00555	−0.04003	0.991182834
M010	Cytosine (3TMS)	13.22 ± 0.01	254	240	170	0.00398	−0.02034	0.995979412
M011	Ergosterol (1TMS)	27.96 ± 0.01	211	364	129	0.00116	−0.01162	0.979552269
M012	Fructose-syn (5TMS)	17.07 ± 0.01	307	217	103	0.00309	−0.00293	0.998573559
M013	Fumaric acid (2TMS)	10.99 ± 0.01	245	147	73	0.0101	−0.0890	0.984258298
M014	Glucose-syn (5TMS)	17.33 ± 0.01	319	205	160	0.00777	−0.04160	0.995802756
M015	Glutamic acid (3TMS)	14.36 ± 0.01	246	147	128	0.00846	−0.05905	0.991560137
M016	Glutamine (3TMS)	16.09 ± 0.01	156	245	73	0.00282	−0.03872	0.952720346
M017	Glycerol (3TMS)	9.89 ± 0.01	205	147	117	0.00465	−0.02928	0.989621885
M018	Glycine (3TMS)	10.38 ± 0.01	174	248	73	0.0217	−0.0472	0.999309039
M019	Glycolic acid (2TMS)	7.04 ± 0.01	205	177	147	0.00119	−0.01314	0.972607162
M020	Guanine (3TMS)	19.59 ± 0.01	352	264	73	0.00705	−0.06942	0.980404182
M021	Histidine (3TMS)	17.61 ± 0.01	154	254	0	0.0133	−0.1708	0.96381778
M022	Inosine (4TMS)	23.31 ± 0.01	217	281	230	0.00624	−0.08974	0.948511774
M023	Isocitric acid (4TMS)	16.55 ± 0.01	245	319	204	0.00604	−0.03097	0.996408844
M024	Isoleucine (2TMS)	10.19 ± 0.01	158	232	218	0.0212	−0.0629	0.998183005
M025	Lactose1 (8TMS)	24.14 ± 0.01	204	361	319	0.0159	−0.1570	0.98100185
M026	Leucine (2TMS)	9.89 ± 0.01	158	232	73	0.0247	−0.0639	0.99895856
M027	Lysine (4TMS)	17.63 ± 0.01	174	317	230	0.0142	−0.0658	0.997051812
M028	Malic acid (3TMS)	12.75 ± 0.01	233	335	147	0.00342	−0.02041	0.993892629
M029	Maltose2 (8TMS)	24.7 ± 0.01	361	204	103	0.00214	−0.02062	0.979669477
M030	Methionine (2TMS)	13.17 ± 0.01	176	293	219	0.0129	−0.0779	0.994154978
M031	Myo-inositol (6TMS)	19.24 ± 0.01	305	265	191	0.0153	−0.0500	0.998677671
M032	Ornithine (4TMS)	16.55 ± 0.01	142	420	174	0.0219	−0.0493	0.998580314
M033	Palmitic acid (1TMS)	18.88 ± 0.01	117	313	129	0.00928	−0.05299	0.992456268
M034	Phenylalanine (2TMS)	14.47 ± 0.01	192	218	73	0.011	−0.0526	0.996722448
M035	Phosphoric acid (3TMS)	9.86 ± 0.01	299	314	211	0.013	−0.1179	0.981330404
M036	Proline (2TMS)	10.27 ± 0.01	142	216	73	0.0242	−0.1219	0.995436881
M037	Putrescine (4TMS)	15.72 ± 0.01	174	214	200	0.043	−0.0501	0.999693324
M038	Pyruvic acid (1metho-oxim 1TMS)	6.64 ± 0.01	174	115	89	0.000672	−0.00155	0.999376913
M039	Raffinose (11TMS)	28.81 ± 0.01	361	437	217	0.0228	−0.1821	0.987840487
M040	Serine (3TMS)	11.09 ± 0.01	204	278	73	0.0145	−0.0565	0.99774593
M041	Stearic acid (1TMS)	20.68 ± 0.01	117	341	145	0.00862	−0.06043	0.98769425
M042	Succinic acid (2TMS)	10.49 ± 0.01	147	129	73	0.0197	−0.0733	0.996924908
M043	Sucrose (8TMS)	23.75 ± 0.01	361	437	217	0.0218	−0.1727	0.988630816
M044	Threonine (3TMS)	11.43 ± 0.01	218	291	117	0.00732	−0.02761	0.997716556
M045	Thymine (2TMS)	11.64 ± 0.01	255	147	113	0.0113	−0.0662	0.99383769
M046	Trehalose (8TMS)	24.55 ± 0.01	191	217	103	0.0167	−0.0683	0.997932624
M047	Tryptophan (2TMS)	20.24 ± 0.01	218	130	100	0.0121	−0.1471	0.970083386
M048	Tyrosine (3TMS)	17.81 ± 0.01	218	280	179	0.0322	−0.1704	0.996105297
M049	Uracil (2TMS)	10.81 ± 0.01	241	99	147	0.00851	−0.04013	0.99612372
M050	Valine (2TMS)	9.09 ± 0.01	144	246	218	0.0202	−0.0626	0.99818831
M051	Xanthine (3TMS)	18.57 ± 0.01	353	368	147	0.004	−0.03673	0.983502896
M052	α-Ketoglutaric acid	13.84 ± 0.01	198	147	89	0.000811	−0.007595	0.980432179
	(1_metho-oxim 2TMS)							

**Table 2:** Result of the addition-recovery test.

ID	Metabolite	Recovery (%)	RSD (%)	ID	Metabolite	Recovery (%)	RSD (%)	ID	Metabolite	Recovery (%)	RSD (%)
M001	4-Aminobutyric acid (3TMS)	93.8	5.6	M019	Glycolic acid (2TMS)	120.2	5.7	M037	Putrescine (4TMS)	89.0	6.9
M002	Aconitic acid (3TMS)	67.1	5.0	M020	Guanine (3TMS)	92.9	6.4	M038	Pyruvic acid (1metho-oxim 1TMS)	114.9	21.1
M003	Adenine (2TMS)	88.1	5.1	M021	Histidine (3TMS)	102.3	5.7	M039	Raffinose (11TMS)	133.1	8.2
M004	Alanine (2TMS)	74.3	16.5	M022	Inosine (4TMS)	98.4	9.0	M040	Serine (3TMS)	88.6	8.7
M005	Asparagine (3TMS)	61.1	6.4	M023	Isocitric acid (4TMS)	90.9	11.0	M041	Stearic acid (1TMS)	81.4	10.0
M006	Aspartic acid (3TMS)	74.8	7.2	M024	Isoleucine (2TMS)	79.1	7.4	M042	Succinic acid (2TMS)	85.1	6.8
M007	Caffeine (0TMS)	96.2	5.5	M025	Lactose1 (8TMS)	109.7	9.7	M043	Sucrose (8TMS)	123.8	9.2
M008	Citric acid (4TMS)	92.7	12.0	M026	Leucine (2TMS)	79.5	10.1	M044	Threonine (3TMS)	92.0	8.5
M009	Cysteine (3TMS)	82.8	3.9	M027	Lysine (4TMS)	120.5	7.0	M045	Thymine (2TMS)	76.3	10.7
M010	Cytosine (3TMS)	92.5	7.6	M028	Malic acid (3TMS)	82.3	5.6	M046	Trehalose (8TMS)	123.0	10.1
M011	Ergosterol (1TMS)	112.5	7.0	M029	Maltose2 (8TMS)	125.5	11.0	M047	Tryptophan (2TMS)	80.0	6.8
M012	Fructose-syn (5TMS)	76.1	21.4	M030	Methionine (2TMS)	86.0	4.2	M048	Tyrosine (3TMS)	111.9	6.7
M013	Fumaric acid (2TMS)	83.9	5.6	M031	Myo-inositol (6TMS)	120.1	7.7	M049	Uracil (2TMS)	79.4	8.2
M014	Glucose-syn (5TMS)	104.0	10.3	M032	Ornithine (4TMS)	99.5	7.7	M050	Valine (2TMS)	68.6	15.1
M015	Glutamic acid (3TMS)	106.7	5.9	M033	Palmitic acid (1TMS)	83.0	9.7	M051	Xanthine (3TMS)	58.3	5.9
M016	Glutamine (3TMS)	129.9	10.0	M034	Phenylalanine (2TMS)	96.7	5.7	M052	α-Ketoglutaric acid (1_metho-oxim 2TMS)	99.1	5.4
M017	Glycerol (3TMS)	110.3	14.2	M035	Phosphoric acid (3TMS)	120.0	11.7				
M018	Glycine (3TMS)	111.7	10.5	M036	Proline (2TMS)	83.1	8.5				

**Table 3:** Comparison of quantitative values of metabolites detected from SRM1950.

ID	Metabolite	RT (min)	This Work	Literature ^a^
		Mean ± SD (*n* = 9)	(mmol/L in Plasma) (*n* = 9)	(mmol/L in Plasma)
M004	Alanine (2TMS)	7.42 ± 0.00	276 ± 21	300 ± 26
M005	Asparagine (3TMS)	14.92 ± 0.00	71.3 ± 1.4	-
M006	Aspartic acid (3TMS)	13.15 ± 0.00	39.5 ± 0.3	-
M008	Citric acid (4TMS)	16.54 ± 0.00	48 ± 2.8	-
M009	Cysteine (3TMS)	13.6 ± 0.00	41.8 ± 0.8	44.3 ± 6.9
M012	Fructose-syn (5TMS)	17.06 ± 0.00	96.9 ± 20.1	-
M014	Glucose-syn (5TMS)	17.33 ± 0.00	4270 ± 366	4560 ± 56
M015	Glutamic acid (3TMS)	14.35 ± 0.00	71.5 ± 3.2	67.4 ± 18
M016	Glutamine (3TMS)	16.08 ± 0.00	284 ± 11	-
M017	Glycerol (3TMS)	9.89 ± 0.00	179 ± 13	-
M018	Glycine (3TMS)	10.38 ± 0.00	143 ± 8	245 ± 16
M021	Histidine (3TMS)	17.6 ± 0.00	93 ± 4.8	72.6 ± 3.6
M024	Isoleucine (2TMS)	10.19 ± 0.00	59.5 ± 2	55.5 ± 3.4
M026	Leucine (2TMS)	9.89 ± 0.00	105 ± 4	100 ± 6
M027	Lysine (4TMS)	17.63 ± 0.00	80.1 ± 11.3	140 ± 14
M030	Methionine (2TMS)	13.17 ± 0.00	40.7 ± 0.4	22.3 ± 1.8
M031	Myo-inositol (6TMS)	19.23 ± 0.00	30.7 ± 1.5	-
M032	Ornithine (4TMS)	16.55 ± 0.00	29 ± 2.7	52.1 ± 2.8
M034	Phenylalanine (2TMS)	14.47 ± 0.00	53.4 ± 1.5	50.8 ± 7
M035	Phosphoric acid (3TMS)	9.87 ± 0.00	275 ± 47	-
M036	Proline (2TMS)	10.27 ± 0.00	158 ± 9	177 ± 9
M038	Pyruvic acid (1metho-oxim 1TMS)	6.62 ± 0.00	283 ± 36	-
M040	Serine (3TMS)	11.09 ± 0.00	73.1 ± 3.8	95.9 ± 4.3
M042	Succinic acid (2TMS)	10.49 ± 0.00	25.6 ± 0.7	-
M044	Threonine (3TMS)	11.43 ± 0.00	103 ± 7	119 ± 6
M047	Tryptophan (2TMS)	20.23 ± 0.00	69 ± 2	-
M048	Tyrosine (3TMS)	17.81 ± 0.00	57.5 ± 2.2	57.3 ± 3
M050	Valine (2TMS)	9.09 ± 0.00	156 ± 10	182 ± 10

^a^ NIST (https://www-s.nist.gov/srmors/certificates/1950.pdf, accessed on 1 February 2021).

## Data Availability

The data presented in this study are available within the article and Supplementary Materials. All tables and figures are original and have not been taken from any publication.

## Supplementary Materials

The following are available online at https://www.mdpi.com/article/10.3390/metabo11040207/s1. Figure S1. Results of calibration curves collection among three different days under DFTPP tuning. Figure S2. Correlation of RPA value and sample amount. Figure S3. Diagram of a robotic platform PAL system. Figure S4. Workflow of the automated sequential derivatization and the injection to GC/MS system using the PAL RTC system. Table S1. Derivatization condition list. Table S2. All detected derivatives and IS. Table S3. Comparative result of derivatization conditions. Table S4. Repeatability and limit of quantification of CCLD. Table S5. Results of GC/MS analysis to optimize the sample amount for SRM 1950.
