# Supplementary material for: Calibration-Curve-Locking Database for Semi-Quantitative Metabolomics by Gas Chromatography/Mass Spectrometry
Source: Metabolites. 2021 Mar 30;11(4):207. doi: 10.3390/metabo11040207 (PMC8065573; doi:10.3390/metabo11040207)
Supplement: Supplementary file 1 [file metabolites-11-00207-s001.pdf]

Supplementary Files

Calibration-Curve-Locking Database for Semi-Quantitative Metabolomics by Gas Chromatography/Mass Spectrometry.

Kosuke Hata[1†], Yuki Soma[1†], Toshiyuki Yamashita[1], Masatomo Takahashi[1], Kuniyo Sugitate[2], Takeshi Serino[2], Hiromi Miyagawa[3], Kenichi Suzuki[3], Kayoko Yamada[4], Takatomo Kawamukai[4], Teruhisa Shiota[4], Yoshihiro Izumi[1*] and Takeshi Bamba[1*]

[1] Division of Metabolomics, Research Center for Transomics Medicine, Medical Institute of Bioregulation, Kyushu University, 3-1-1 Maidashi, Higashi-ku, Fukuoka 812-8582, Japan; k-hata@bioreg.kyushu-u.ac.jp (K.H.); y-soma@bioreg.kyushu-u.ac.jp (Y.S.); toshiyuki_yamashita@bioreg.kyushu-u.ac.jp (T.Y.); m-takahashi@bioreg.kyushu-u.ac.jp (M.T.); izumi@bioreg.kyushu-u.ac.jp (Y.I.); bamba@bioreg.kyushu-u.ac.jp (T.B.);

[2] Agilent Technologies Japan Ltd., 9-1, Takakuramachi, Hachioji-shi, Tokyo, Japan; kuniyo_sugitate@agilent.com (K.S.); takeshi_serino@agilent.com (T.S.);

[3] GL Sciences Inc., Tokyo, Japan, 6-22-1, Nishi-Shinjuku, Shinjuku-ku, Tokyo; h.miyagawa@gls.co.jp (H.M.); suzuki@gls.co.jp (K.S.);

[4] AMR Inc., Tokyo, Japan, 2-13-18 Nakane, Meguro-ku, Tokyo, Japan; k-yamada@amr-inc.co.jp (K.Y.); t-kawamukai@amr-inc.co.jp (T.K.); t-shiota@amr-inc.co.jp (T.S.);

[*] Correspondence: izumi@bioreg.kyushu-u.ac.jp; bamba@bioreg.kyushu-u.ac.jp; Tel.: +81-92-802-4729

[†] These authors contributed equally to this work

1. Supplementary Figures

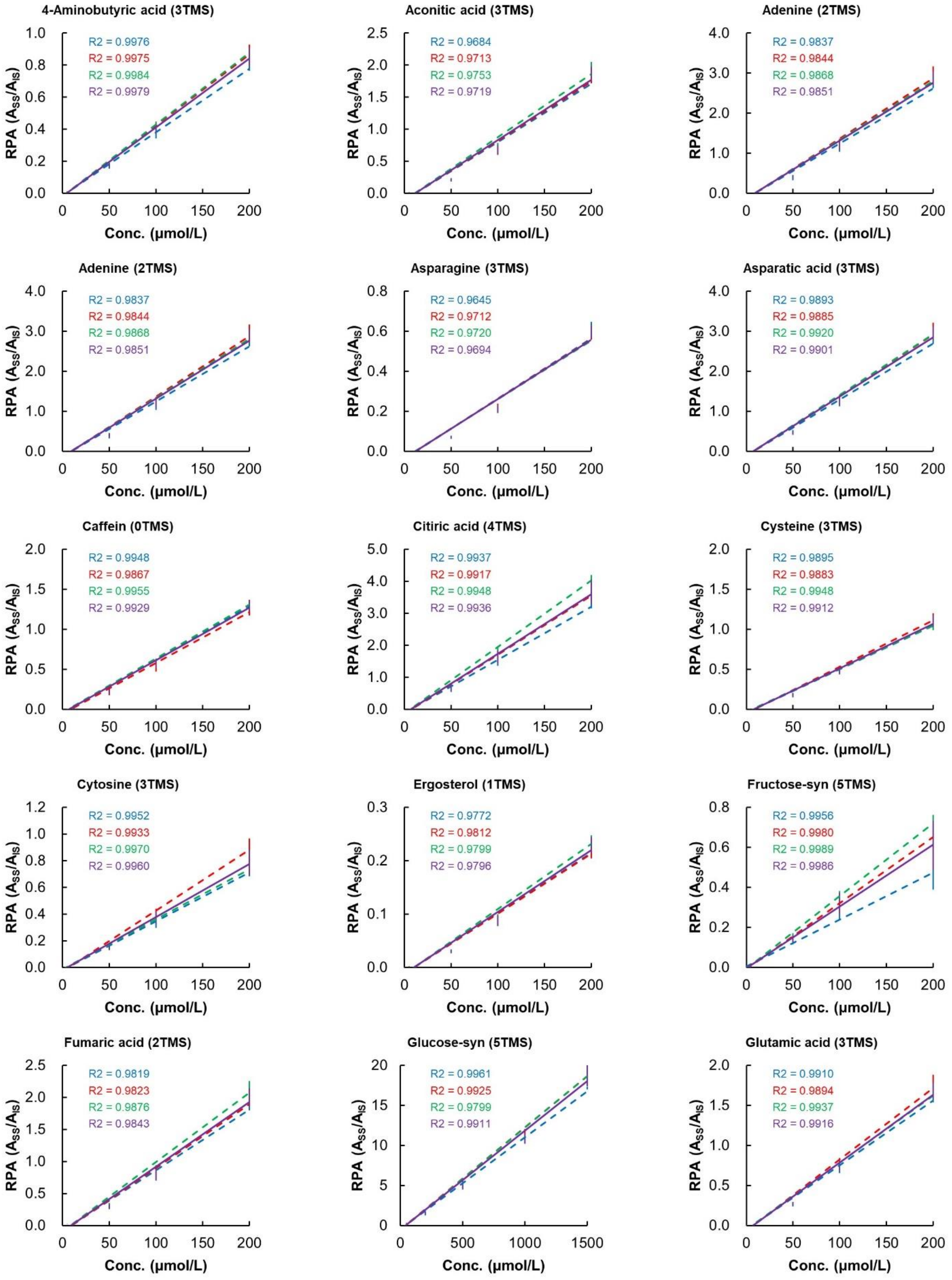

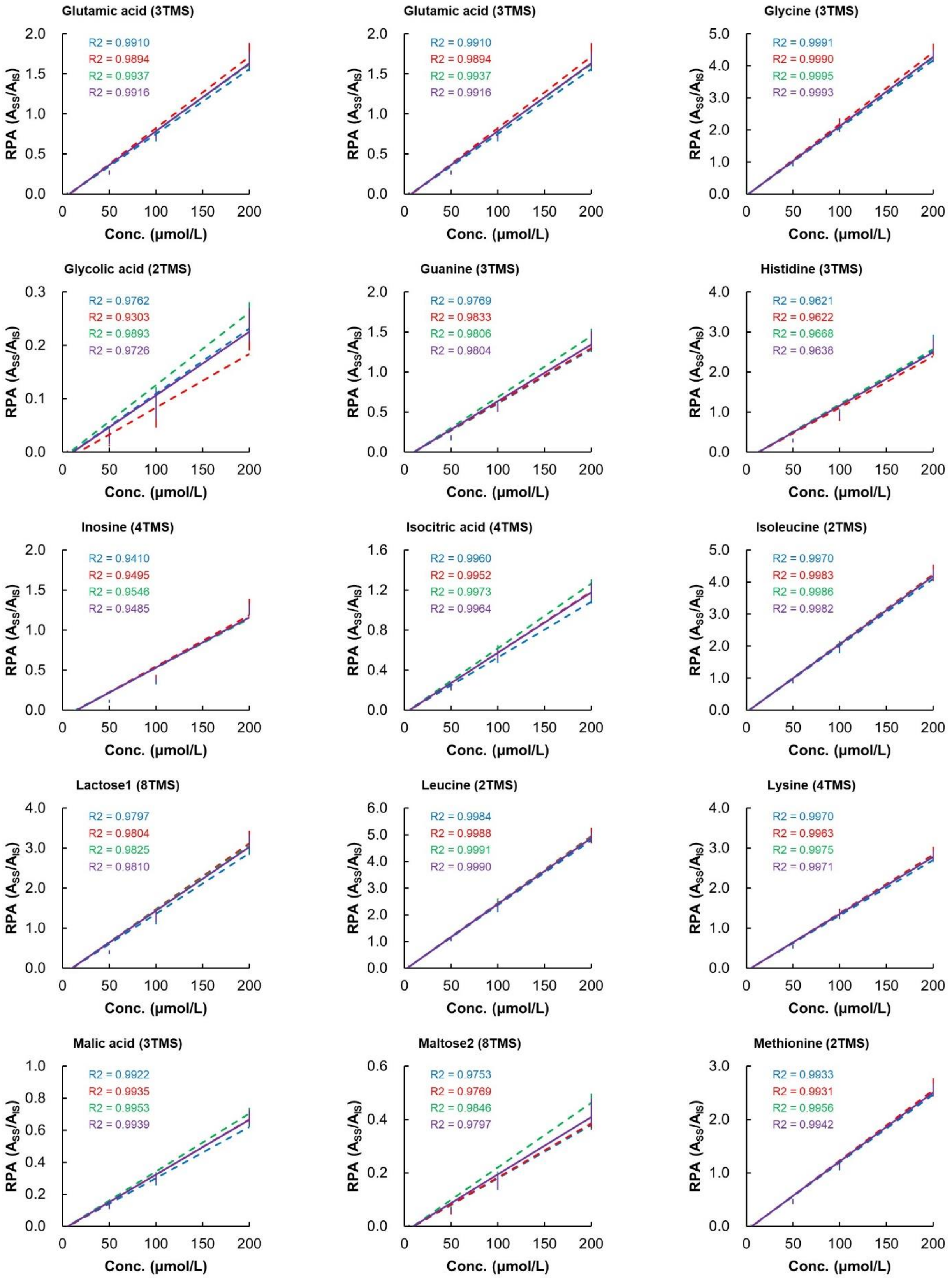
Glutamic acid (3TMS)
R2 = 0.9910
R2 = 0.9894
R2 = 0.9937
R2 = 0.9916
Glutamic acid (3TMS)
R2 = 0.9910
R2 = 0.9894
R2 = 0.9937
R2 = 0.9916
Glycine (3TMS)
R2 = 0.9991
R2 = 0.9990
R2 = 0.9995
R2 = 0.9993
Glycolic acid (2TMS)
R2 = 0.9762
R2 = 0.9303
R2 = 0.9893
R2 = 0.9726
Guanine (3TMS)
R2 = 0.9769
R2 = 0.9833
R2 = 0.9806
R2 = 0.9804
Histidine (3TMS)
R2 = 0.9621
R2 = 0.9622
R2 = 0.9668
R2 = 0.9638
Inosine (4TMS)
R2 = 0.9410
R2 = 0.9495
R2 = 0.9546
R2 = 0.9485
Isocitric acid (4TMS)
R2 = 0.9960
R2 = 0.9952
R2 = 0.9973
R2 = 0.9964
Isoleucine (2TMS)
R2 = 0.9970
R2 = 0.9983
R2 = 0.9986
R2 = 0.9982
Lactose1 (8TMS)
R2 = 0.9797
R2 = 0.9804
R2 = 0.9825
R2 = 0.9810
Leucine (2TMS)
R2 = 0.9984
R2 = 0.9988
R2 = 0.9991
R2 = 0.9990
Lysine (4TMS)
R2 = 0.9970
R2 = 0.9963
R2 = 0.9975
R2 = 0.9971
Malic acid (3TMS)
R2 = 0.9922
R2 = 0.9935
R2 = 0.9953
R2 = 0.9939
Maltose2 (8TMS)
R2 = 0.9753
R2 = 0.9769
R2 = 0.9846
R2 = 0.9797
Methionine (2TMS)
R2 = 0.9933
R2 = 0.9931
R2 = 0.9956
R2 = 0.9942

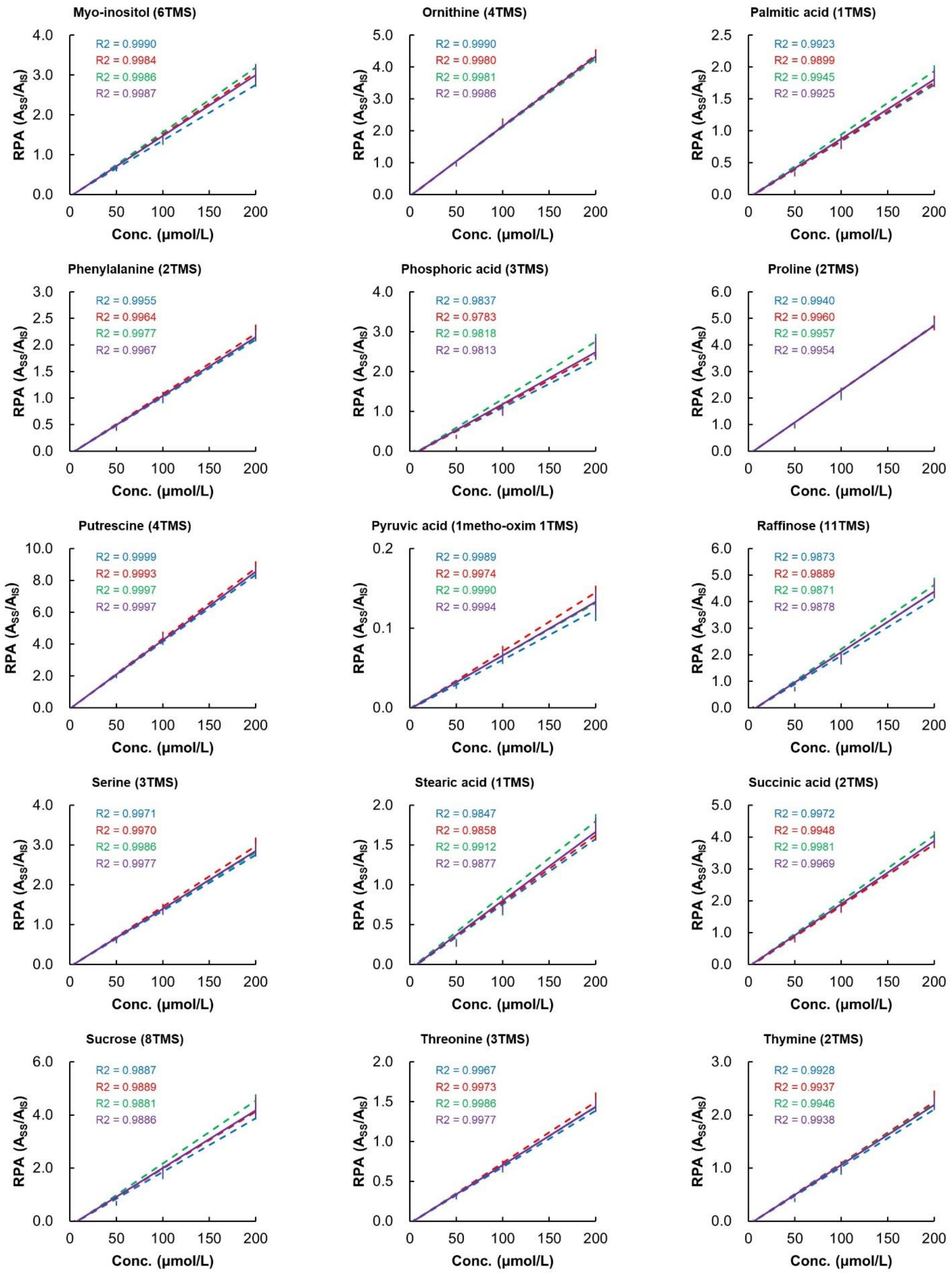
Myo-inositol (6TMS)
R2 = 0.9990
R2 = 0.9984
R2 = 0.9986
R2 = 0.9987
Ornithine (4TMS)
R2 = 0.9990
R2 = 0.9980
R2 = 0.9981
R2 = 0.9986
Palmitic acid (1TMS)
R2 = 0.9923
R2 = 0.9899
R2 = 0.9945
R2 = 0.9925
Phenylalanine (2TMS)
R2 = 0.9955
R2 = 0.9964
R2 = 0.9977
R2 = 0.9967
Phosphoric acid (3TMS)
R2 = 0.9837
R2 = 0.9783
R2 = 0.9818
R2 = 0.9813
Proline (2TMS)
R2 = 0.9940
R2 = 0.9960
R2 = 0.9957
R2 = 0.9954
Putrescine (4TMS)
R2 = 0.9999
R2 = 0.9993
R2 = 0.9997
R2 = 0.9997
Pyruvic acid (1metho-oxim 1TMS)
R2 = 0.9989
R2 = 0.9974
R2 = 0.9990
R2 = 0.9994
Raffinose (11TMS)
R2 = 0.9873
R2 = 0.9889
R2 = 0.9871
R2 = 0.9878
Serine (3TMS)
R2 = 0.9971
R2 = 0.9970
R2 = 0.9986
R2 = 0.9977
Stearic acid (1TMS)
R2 = 0.9847
R2 = 0.9858
R2 = 0.9912
R2 = 0.9877
Succinic acid (2TMS)
R2 = 0.9972
R2 = 0.9948
R2 = 0.9981
R2 = 0.9969
Sucrose (8TMS)
R2 = 0.9887
R2 = 0.9889
R2 = 0.9881
R2 = 0.9886
Threonine (3TMS)
R2 = 0.9967
R2 = 0.9973
R2 = 0.9986
R2 = 0.9977
Thymine (2TMS)
R2 = 0.9928
R2 = 0.9937
R2 = 0.9946
R2 = 0.9938
RPA (A_SS/A_IS)
Conc. (μmol/L)

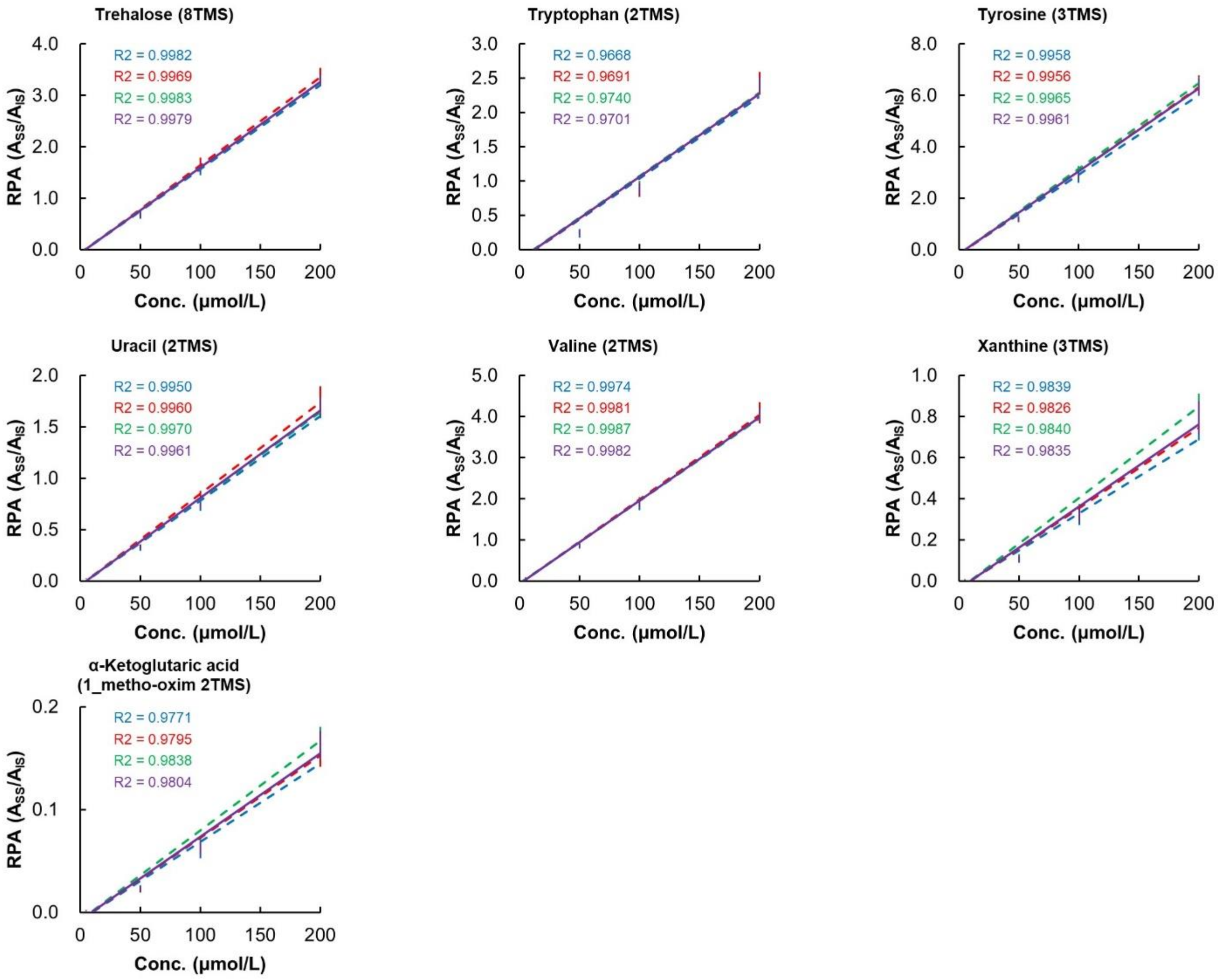

Figure S1. Results of calibration curves collection among three deferent days under DFTPP tuning. RPA was plotted versus amount ratio of standard substrate to IS. Each blue, red, and green dash line represents the result of day 1, day 2 and day 3, respectively (*n*=3 for each day). Purple line represents the overall average of three days (*n*=9). Error bars represent the standard deviation.

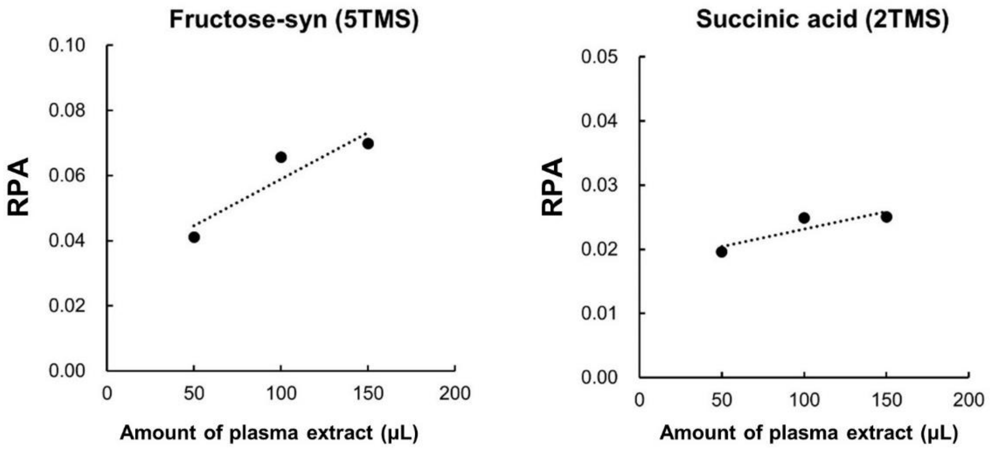

Figure S2. Correlation of RPA value and sample amount. When 50, 100 and 150 μL of plasma extracts were analyzed based on method E, linear response was observed between 50~150 μL for each target metabolites excepting fructose and succinic acid. Regarding the above two metabolites, linear response was observed between 50~100 μL.

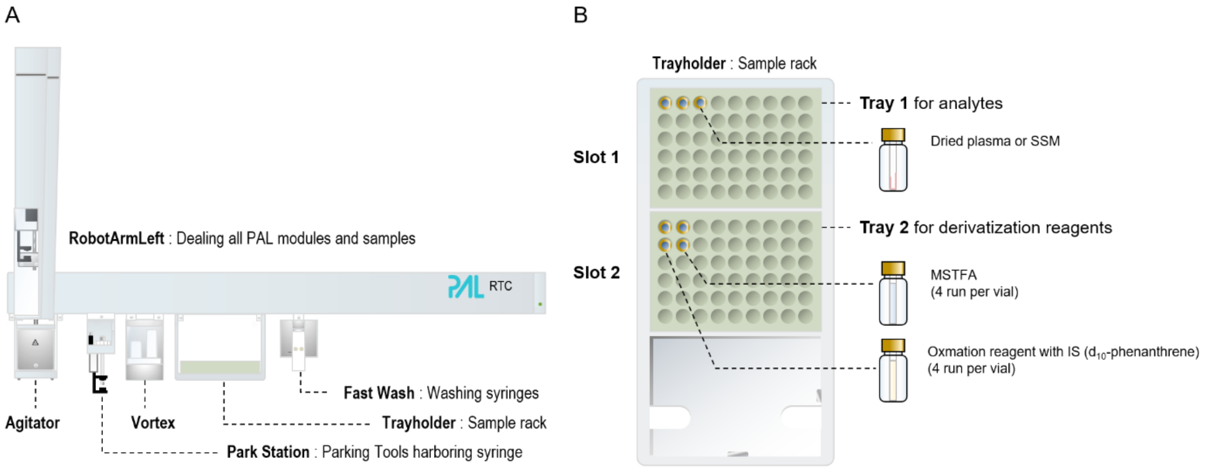

Figure S3. Diagram of a robotic platform PAL system. (A) Configuration of PAL RTC system. (B) The samples and reagents in tray holder.

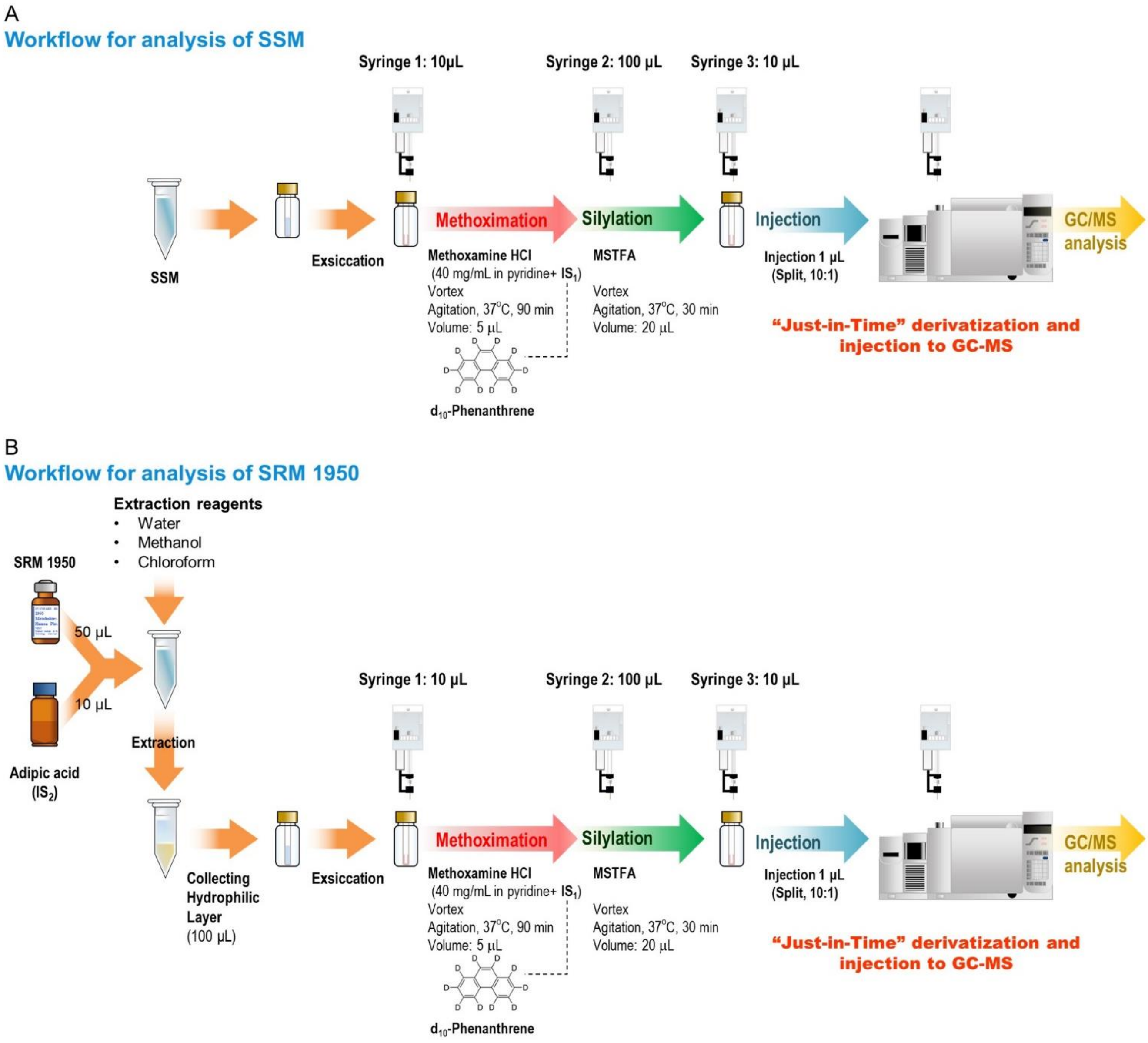

Figure S4. Workflow of the automated sequential derivatization and the injection to GC/MS system using the PAL RTC system for the analysis of SSMs (A) and for the analysis of plasma sample (B).

2. Supplementary Tables.

Table S1. Derivatization condition list.

**Derivatization conditions**		**Control** [a]	**A**	**B**	**C**	**D**	**E**
SSM	Stock conc. (µmol/L)	200	200	200	200	100	50
	Addition vol. (µL)	100	100	100	100	**100**	**100**
	Addition amount (µmol)	0.02	0.02	0.02	0.02	0.01	0.005
	Final conc. (µmol/L)	200	200	200	200	200	200
IS_1 (d_{10}-phenanthrene)	Stock conc. (µg/mL)	100	100	100	100	100	**50**
	Addition vol. (µL)	10	10	10	10	5	5
	Addition amount (ng)	1000	1000	1000	1000	500	250
	Final conc. (µg/µL)	10	10	10	10	10	10
Oximation	Reaction temp. (°C)	30	30	**37**	**50**	**37**	**37**
	Reaction time (min)	90	90	90	90	90	90
	Reagent conc. (mg/mL)	40	40	40	40	40	40
	Reagent vol. (µL)	10	10	10	10	**5**	**5**
Silylation	Reaction temp. (°C)	37	**30**	37	**50**	37	37
	Reaction time (min)	30	30	30	30	30	30
	Reagent vol. (µL)	90	90	90	90	**45**	**20**
Over all	Final vol. (µL)	100	100	100	100	**50**	**25**

[a] Tobias Kind et al, Anal. Chem., 2009, 81, 10038–10048.

The part where the bold font indicates difference from the control condition

Table S2. All detected derivatives and IS

Seq. No.	ID	**Metabolite**	**RT (min)** [a] **mean ± SD (n = 9)**	**Quantifier (m/z)**	**Qualifier 1 (m/z)**	**Qualifier 2 (m/z)**
1	M001	4-Aminobutyric acid (3TMS)	13.31 ± 0.01	304	174	147
2	M002	Aconitic acid (3TMS)	15.81 ± 0.01	147	375	229
3	M003	Adenine (2TMS)	17.12 ± 0.01	264	279	192
4	M004	Alanine (2TMS)	7.43 ± 0.01	116	190	147
5	M004	Alanine (3TMS)	11.11 ± 0.01	262	188	100
6	M005	Asparagine (2TMS)	14.43 ± 0.01	188	303	216
7	M005	Asparagine (3TMS)	14.93 ± 0.01	116	231	132
8	M006	Aspartic acid (2TMS)	11.96 ± 0.01	160	130	117
9	M006	Aspartic acid (3TMS)	13.16 ± 0.01	232	218	100
10	M007	Caffeine (0TMS)	17.01 ± 0.01	194	109	67
11	M008	Citric acid (4TMS)	16.55 ± 0.01	273	465	347
12	M009	Cysteine (2TMS)	12.57 ± 0.01	148	250	132
13	M009	Cysteine (3TMS)	13.6 ± 0.01	218	220	100
14	M009	Cystine (4TMS)	21.07 ± 0.01	218	411	146

15	M010	Cytosine (1Metho-oxim, 2TMS)	13.49 ± 0.01	285	270	240
16	M010	Cytosine (3TMS)	13.22 ± 0.01	254	240	170
17	M011	Ergosterol (1TMS)	27.96 ± 0.01	211	364	129
18	M012	Fructose-anti (5TMS)	17.16 ± 0.01	307	217	103
19	M012	Fructose-syn (5TMS)	17.07 ± 0.01	307	217	103
20	M013	Fumaric acid (2TMS)	10.99 ± 0.01	245	147	73
21	M014	Glucopyranose (5TMS)	18.12 ± 0.01	204	191	147
22	M014	Glucose-anti (5TMS)	17.53 ± 0.01	319	205	160
23	M014	Glucose-syn (5TMS)	17.33 ± 0.01	319	205	160
24	M015	Glutamic acid (2TMS)	13.32 ± 0.01	84	158	56
25	M015	Glutamic acid (3TMS)	14.36 ± 0.01	246	147	128
26	M016	Glutamine (2TMS)	14.02 ± 0.01	227	334	301
27	M016	Glutamine (3TMS)	16.09 ± 0.01	156	245	73
28	M017	Glycerol (3TMS)	9.89 ± 0.01	205	147	117
29	M018	Glycine (3TMS)	10.38 ± 0.01	174	248	73
30	M019	Glycolic acid (2TMS)	7.04 ± 0.01	205	177	147
31	M020	Guanine (3TMS)	19.59 ± 0.01	352	264	73
32	M021	Histidine (3TMS)	17.61 ± 0.01	154	254	0
33	M022	Inosine (4TMS)	23.31 ± 0.01	217	281	230
34	M023	Isocitric acid (4TMS)	16.55 ± 0.01	245	319	204
35	M024	Isoleucine (1TMS)	8.58 ± 0.01	86	188	170
36	M024	Isoleucine (2TMS)	10.19 ± 0.01	158	232	218
37	M025	Lactose1 (8TMS)	24.14 ± 0.01	204	361	319
38	M025	Lactose2 (8TMS)	24.27 ± 0.01	204	361	319
39	M026	Leucine (1TMS)	8.26 ± 0.01	86	146	75
40	M026	Leucine (2TMS)	9.89 ± 0.01	158	232	73
41	M027	Lysine (3TMS)	17.11 ± 0.04	200	362	258
42	M027	Lysine (4TMS)	17.63 ± 0.01	174	317	230
43	M028	Malic acid (3TMS)	12.75 ± 0.01	233	335	147
44	M029	Maltose1 (8TMS)	24.5 ± 0.01	204	319	103
45	M029	Maltose2 (8TMS)	24.7 ± 0.01	361	204	103
46	M030	Methionine (1TMS)	11.83 ± 0.01	104	178	61
47	M030	Methionine (2TMS)	13.17 ± 0.01	176	293	219
48	M031	Myo-inositol (6TMS)	19.24 ± 0.01	305	265	191
49	M032	Ornithine (3TMS)	14.36 ± 0.01	142	348	73
50	M032	Ornithine (4TMS)	16.55 ± 0.01	142	420	174
51	M033	Palmitic acid (1TMS)	18.88 ± 0.01	117	313	129
52	M034	Phenylalanine (1TMS)	13.57 ± 0.01	120	146	91
53	M034	Phenylalanine (2TMS)	14.47 ± 0.01	192	218	73
54	M035	Phosphoric acid (3TMS)	9.86 ± 0.01	299	314	211

55	M036	Proline (1TMS)	8.55 ± 0.01	172	103	68
56	M036	Proline (2TMS)	10.27 ± 0.01	142	216	73
57	M037	Putrescine (4TMS)	15.72 ± 0.01	174	214	200
58	M037	Pyroglutamic acid (2TMS)	13.21 ± 0.01	156	258	230
59	M038	Pyruvic acid (1metho-oxim 1TMS)	6.64 ± 0.01	174	115	89
60	M039	Raffinose (11TMS)	28.81 ± 0.01	361	437	217
61	M040	Serine (2TMS)	9.69 ± 0.01	219	132	116
62	M040	Serine (3TMS)	11.09 ± 0.01	204	278	73
63	M041	Stearic acid (1TMS)	20.68 ± 0.01	117	341	145
64	M042	Succinic acid (2TMS)	10.49 ± 0.01	147	129	73
65	M043	Sucrose (8TMS)	23.75 ± 0.01	361	437	217
66	M044	Threonine (2TMS)	10.23 ± 0.03	117	219	130
67	M044	Threonine (3TMS)	11.43 ± 0.01	218	291	117
68	M045	Thymine (2TMS)	11.64 ± 0.01	255	147	113
69	M046	Trehalose (8TMS)	24.55 ± 0.01	191	217	103
70	M047	Tryptophan (2TMS)	20.24 ± 0.01	218	130	100
71	M047	Tryptophan (3TMS)	20.41 ± 0.01	202	291	73
72	M048	Tyrosine (2TMS)	17.31 ± 0.01	179	208	0
73	M048	Tyrosine (3TMS)	17.81 ± 0.01	218	280	179
74	M049	Uracil (2TMS)	10.81 ± 0.01	241	99	147
75	M050	Valine (1TMS)	7.26 ± 0.01	72	174	156
76	M050	Valine (2TMS)	9.09 ± 0.01	144	246	218
77	M051	Xanthine (3TMS)	18.57 ± 0.01	353	368	147
78	M052	α-Ketoglutaric acid (1metho-oxim, 2TMS)	13.84 ± 0.01	198	147	89
79	IS1	d_{10}-Phenanthrene	16.46 ± 0.01	188	-	-
80	IS2	Adipic acid (2TMS)	11.196 ± 4.5604	111	275	172

[a] RT was obtained with 200 μmol/L of SSM.

Table S3. Comparative result of derivatization conditions

Seq. No.	ID	Compound name	Comparison of RPA				
			A/Control	B/Control	C/Control	D/Control	E/Control
1	M001	4-Aminobutyric acid (3TMS)	1.0	1.0	1.0	1.1	1.1
2	M002	Aconitic acid (3TMS)	0.9	1.0	1.0	0.9	1.0
3	M003	Adenine (2TMS)	1.0	1.0	1.0	1.1	1.1
4	M004	Alanine (2TMS) main peak	1.0	1.0	1.0	0.9	1.0
5	M004	Alanine (3TMS)	0.9	1.1	1.1	1.3	1.6
6	M005	Asparagine (2TMS)	1.2	0.9	1.1	0.8	0.6
7	M005	Asparagine (3TMS) main peak	1.0	1.1	0.9	1.2	1.2
8	M006	Aspartic acid (2TMS)	0.9	1.2	0.9	1.5	2.2
9	M006	Aspartic acid (3TMS) main peak	0.9	1.0	1.0	1.1	1.1
10	M007	Caffeine (0TMS)	1.0	1.0	1.0	1.0	1.0
11	M008	Citric acid (4TMS)	1.0	1.0	1.0	1.1	1.1
12	M009	Cysteine (2TMS)	0.8	1.4	1.1	1.6	2.5
13	M009	Cysteine (3TMS) main peak	1.0	1.2	1.3	1.4	1.3
14	M009	Cystine (4TMS)	1.0	1.2	1.7	1.2	1.1
15	M010	Cytosine (1Metho-oxim, 2TMS)	1.1	1.6	3.8	1.9	1.7
16	M010	Cytosine (3TMS) main peak	0.9	0.9	0.5	0.9	1.1
17	M011	Ergosterol (1TMS)	0.9	1.0	1.0	1.0	1.0
18	M012	Fructose-anti (5TMS)	1.0	0.8	0.9	1.2	1.2
19	M012	Fructose-syn (5TMS) main peak	1.1	0.8	0.8	1.2	1.3
20	M013	Fumaric acid (2TMS)	0.9	1.0	0.8	1.0	1.0
21	M014	Glucopyranose (5TMS)	0.6	0.2	0.1	0.2	0.1
22	M014	Glucose-anti (5TMS)	1.1	1.0	1.0	1.2	1.2
23	M014	Glucose-syn (5TMS) main peak	1.0	1.0	1.0	1.2	1.2
24	M015	Glutamic acid (2TMS)	0.9	1.1	0.9	1.5	2.0
25	M015	Glutamic acid (3TMS) main peak	1.1	1.0	0.9	1.1	1.2
26	M016	Glutamine (2TMS)	0.9	1.0	0.9	1.5	1.5
27	M016	Glutamine (3TMS) main peak	1.0	0.9	0.5	1.1	1.2
28	M017	Glycerol (3TMS)	1.0	1.0	1.0	1.0	1.0
29	M018	Glycine (3TMS)	1.0	1.0	1.0	1.0	1.0
30	M019	Glycolic acid (2TMS)	1.0	1.0	1.0	1.0	1.0
31	M020	Guanine (3TMS)	1.0	1.1	1.0	1.1	1.1
32	M021	Histidine (3TMS)	1.1	1.1	1.0	1.0	1.0
33	M022	Inosine (4TMS)	1.0	1.1	1.0	1.0	1.1
34	M023	Isocitric acid (4TMS)	1.0	1.0	1.0	1.1	1.1
35	M024	Isoleucine (1TMS)	1.0	1.1	0.9	1.1	0.8
36	M024	Isoleucine (2TMS) main peak	1.0	1.0	1.0	1.0	1.1
37	M025	Lactose1 (8TMS) main peak	0.9	1.0	1.0	1.1	1.1
38	M025	Lactose2 (8TMS)	1.0	1.0	1.0	1.1	1.1
39	M026	L-leucine (1TMS)	1.0	1.1	0.9	1.2	0.9
40	M026	L-leucine (2TMS) main peak	1.0	1.0	1.0	1.0	1.1
41	M027	Lysine (3TMS)	1.0	0.8	0.8	1.3	1.3
42	M027	Lysine (4TMS) main peak	1.0	1.0	1.0	1.0	1.0
43	M028	Malic acid (3TMS)	1.0	1.0	1.0	1.0	1.0
44	M029	Maltose1 (8TMS)	1.1	1.1	1.2	1.2	1.3
45	M029	Maltose2 (8TMS) main peak	1.1	1.1	1.1	1.2	1.3
46	M030	Methionine (1TMS)	1.0	1.2	0.9	1.5	2.2
47	M030	Methionine (2TMS) main peak	1.0	1.0	1.0	1.0	1.0
48	M031	Myo-inositol (6TMS)	1.0	1.0	1.0	1.1	1.1
49	M032	Ornithine (3TMS)	1.3	1.1	1.0	1.0	1.1
50	M032	Ornithine (4TMS) main peak	1.0	1.0	1.0	1.1	1.1
51	M033	Palmitic acid (1TMS)	0.9	1.0	1.0	1.0	1.0
52	M034	Phenylalanine (1TMS)	0.9	1.3	0.9	1.6	2.5
53	M034	Phenylalanine (2TMS) main peak	1.0	1.0	1.0	1.0	1.0
54	M035	Phosphoric acid (3TMS)	1.0	1.1	1.0	1.2	1.1
55	M036	Proline (1TMS)	1.1	1.1	0.9	1.1	0.8
56	M036	Proline (2TMS) main peak	1.0	1.0	1.0	1.0	1.1
57	M037	Putrescine (4TMS)	1.0	1.0	1.0	1.0	1.0
58	M015	Pyroglutamic acid (2TMS)	0.9	1.3	1.9	1.2	1.2
59	M038	Pyruvic acid (1metho-oxim, 1TMS)	0.9	1.0	0.9	1.0	1.0
60	M039	Raffinose (11TMS)	1.0	1.0	1.0	1.3	1.3

61	M040	Serine (2TMS)	1.1	1.4	0.9	1.6	1.8
62	M040	Serine (3TMS) main peak	1.0	1.0	1.0	1.0	1.1
63	M041	Stearic acid (1TMS)	0.9	1.0	1.0	1.1	1.1
64	M042	Succinic acid (2TMS)	0.9	1.0	1.0	0.9	1.0
65	M043	Sucrose (8TMS)	1.0	1.0	1.0	1.2	1.2
66	M044	Threonine (2TMS)	1.0	1.2	0.9	1.2	1.5
67	M044	Threonine (3TMS) main peak	1.0	1.0	1.0	1.1	1.1
68	M045	Thymine (2TMS)	1.0	1.0	1.0	1.1	1.2
69	M046	Trehalose (8TMS)	0.9	1.0	1.0	1.0	1.0
70	M047	Tryptophan (2TMS) main peak	1.0	1.0	1.0	1.0	1.0
71	M047	Tryptophan (3TMS)	0.7	0.9	1.6	1.7	1.3
72	M048	Tyrosine (2TMS)	0.9	1.1	0.9	2.1	2.7
73	M048	Tyrosine (3TMS) main peak	0.9	1.0	1.0	1.1	1.1
74	M049	Uracil (2TMS)	1.0	1.0	1.0	1.1	1.1
75	M050	Valine (1TMS)	1.0	1.1	0.9	1.1	1.1
76	M050	Valine (2TMS)	1.0	1.0	1.0	1.0	1.0
77	M051	Xanthine (3TMS)	1.0	1.0	1.0	1.2	1.3
78	M052	α-Ketoglutaric acid (1metho-oxim, 2TMS)	1.0	1.5	2.0	1.5	1.5

Table S4. Repeatability and LOQ of CCLD

ID	Metabolite	RT (min)	Repeatability of slope of calibration curves, RSD (%)				LOQ (μmol/L)
			Day 1 (*n* = 3)	Day 2 (*n* = 3)	Day 3 (*n* = 3)	Average (*n* = 9)	
1	4-Aminobutyric acid (3TMS)	13.31 ± 0.01	0.0	0.0	0.0	0.0	5
2	Aconitic acid (3TMS)	15.80 ± 0.01	0.0	11.1	0.0	11.1	5
3	Adenine (2TMS)	17.12 ± 0.01	7.1	6.7	0.0	7.1	5
4	Alanine (2TMS)	7.43 ± 0.01	5.6	5.3	0.0	5.6	5
5	Asparagine (3TMS)	14.93 ± 0.01	0.0	0.0	0.0	0.0	5
6	Aspartic acid (3TMS)	13.16 ± 0.01	7.1	6.7	0.0	6.7	5
7	Caffeine (0TMS)	17.00 ± 0.01	0.0	0.0	0.0	0.0	5
8	Citric acid (4TMS)	16.55 ± 0.01	5.9	5.6	0.0	10.5	5
9	Cysteine (3TMS)	13.60 ± 0.01	0.0	0.0	0.0	0.0	5
10	Cytosine (3TMS)	13.22 ± 0.01	0.0	0.0	0.0	0.0	5
11	Ergosterol (1TMS)	27.95 ± 0.01	0.0	0.0	0.0	0.0	50
12	Fructose-syn (5TMS)	17.07 ± 0.01	0.0	0.0	0.0	33.3	5
13	Fumaric acid (2TMS)	10.99 ± 0.01	0.0	0.0	9.1	10.0	5
14	Glucose-syn (5TMS)	17.33 ± 0.01	0.0	7.7	7.1	7.7	5
15	Glutamic acid (3TMS)	14.36 ± 0.01	0.0	11.1	0.0	0.0	5
16	Glutamine (3TMS)	16.09 ± 0.01	0.0	0.0	0.0	0.0	5
17	Glycerol (3TMS)	9.89 ± 0.01	0.0	0.0	0.0	0.0	5
18	Glycine (3TMS)	10.38 ± 0.01	4.8	4.5	0.0	4.5	50
19	Glycolic acid (2TMS)	7.03 ± 0.01	0.0	0.0	0.0	0.0	50
20	Guanine (3TMS)	19.59 ± 0.01	0.0	0.0	0.0	14.3	5
21	Histidine (3TMS)	17.61 ± 0.01	7.7	7.7	0.0	7.7	5
22	Inosine (4TMS)	23.3 ± 0.01	0.0	0.0	0.0	0.0	5
23	Isocitric acid (4TMS)	16.55 ± 0.01	0.0	0.0	0.0	0.0	5
24	Isoleucine (2TMS)	10.19 ± 0.01	4.8	4.5	0.0	4.8	5
25	Lactose1 (8TMS)	24.14 ± 0.01	6.7	6.3	0.0	6.3	5
26	Leucine (2TMS)	9.89 ± 0.01	4.2	4.0	4.0	4.0	5

27	Lysine (4TMS)	17.63 ± 0.01	0.0	6.7	0.0	7.1	5
28	Malic acid (3TMS)	12.75 ± 0.01	0.0	0.0	0.0	0.0	5
29	Maltose2 (8TMS)	24.70 ± 0.01	0.0	0.0	0.0	0.0	5
30	Methionine (2TMS)	13.17 ± 0.01	7.7	7.7	0.0	7.7	5
31	Myo-inositol (6TMS)	19.24 ± 0.01	0.0	6.3	0.0	6.7	5
32	Ornithine (4TMS)	16.55 ± 0.01	4.5	4.5	0.0	4.5	5
33	Palmitic acid (1TMS)	18.88 ± 0.01	0.0	11.1	0.0	11.1	5
34	Phenylalanine (2TMS)	14.47 ± 0.01	0.0	9.1	0.0	0.0	5
35	Phosphoric acid (3TMS)	9.86 ± 0.01	8.3	7.7	0.0	7.7	5
36	Proline (2TMS)	10.27 ± 0.01	4.2	4.2	4.2	4.2	5
37	Putrescine (4TMS)	15.72 ± 0.01	2.4	4.5	2.3	4.7	5
38	Pyruvic acid (1metho-oxim, 1TMS)	6.63 ± 0.01	0.0	0.0	0.0	0.0	50
39	Raffinose (11TMS)	28.81 ± 0.01	4.8	8.7	4.2	8.7	5
40	Serine (3TMS)	11.09 ± 0.01	0.0	6.7	0.0	7.1	5
41	Stearic acid (1TMS)	20.68 ± 0.01	0.0	12.5	0.0	11.1	5
42	Succinic acid (2TMS)	10.49 ± 0.01	5.3	5.3	0.0	5.0	5
43	Sucrose (8TMS)	23.75 ± 0.01	5.0	4.8	0.0	9.1	5
44	Threonine (3TMS)	11.43 ± 0.01	0.0	0.0	0.0	0.0	5
45	Thymine (2TMS)	11.64 ± 0.01	0.0	8.3	0.0	9.1	5
46	Trehalose (8TMS)	24.55 ± 0.01	0.0	5.9	0.0	5.9	5
47	Tryptophan (2TMS)	20.24 ± 0.01	8.3	8.3	0.0	8.3	5
48	Tyrosine (3TMS)	17.81 ± 0.01	3.2	6.1	3.0	3.1	5
49	Uracil (2TMS)	10.81 ± 0.01	0.0	11.1	0.0	0.0	5
50	Valine (2TMS)	9.09 ± 0.01	5.0	4.8	0.0	5.0	5
51	Xanthine (3TMS)	18.57 ± 0.01	0.0	0.0	0.0	0.0	5
52	α-Ketoglutaric acid (1metho-oxim, 2TMS)	13.84 ± 0.01	0.0	0.0	0.0	0.0	50

Table S5. Optimization of amount of plasma extract for CCLD-based GC/MS quantification

ID	Metabolite	RPA (mean ± SD, *n* = 3)			R^2
		Amount of plasma extract (upper phase)			
		50 μL	100 μL	150 μL	
4	Alanine (2TMS)	0.397 ± 0.023	0.735 ± 0.036	1.001 ± 0.069	0.995
5	Asparagine (3TMS)	0.003 ± 0	0.006 ± 0	0.007 ± 0	0.973
8	Citric acid (4TMS)	0.028 ± 0.001	0.056 ± 0.002	0.075 ± 0.002	0.991
9	Cysteine (3TMS)	0.003 ± 0.001	0.005 ± 0.001	0.007 ± 0	0.964
12	Fructose-syn (5TMS)	0.041 ± 0.006	0.066 ± 0.017	0.07 ± 0.032	0.855
14	Glucose-syn (5TMS)	3.602 ± 0.282	8.725 ± 0.601	12.92 ± 0.71	0.997
15	Glutamic acid (3TMS)	0.029 ± 0.002	0.051 ± 0.002	0.065 ± 0.005	0.984
16	Glutamine (3TMS)	0.052 ± 0.005	0.092 ± 0.011	0.139 ± 0.004	0.998
17	Glycerol (3TMS)	0.073 ± 0.005	0.126 ± 0.006	0.164 ± 0.011	0.991
18	Glycine (3TMS)	0.303 ± 0.057	0.486 ± 0.024	0.562 ± 0.025	0.946
21	Histidine (3TMS)	0.039 ± 0.005	0.078 ± 0.008	0.109 ± 0.014	0.996
24	Isoleucine (2TMS)	0.089 ± 0.008	0.172 ± 0.012	0.237 ± 0.02	0.995
26	Leucine (2TMS)	0.204 ± 0.015	0.403 ± 0.027	0.553 ± 0.042	0.993
27	Lysine (4TMS)	0.086 ± 0.017	0.171 ± 0.03	0.23 ± 0.032	0.989
30	Methionine (2TMS)	0.013 ± 0.001	0.024 ± 0.001	0.032 ± 0.003	0.996
31	Myo-inositol (6TMS)	0.018 ± 0.001	0.036 ± 0.001	0.051 ± 0.003	0.997
32	Ornithine (4TMS)	0.024 ± 0.018	0.054 ± 0.044	0.1 ± 0.014	0.986
34	Phenylalanine (2TMS)	0.028 ± 0.003	0.059 ± 0.004	0.086 ± 0.006	0.998
35	Phosphoric acid (3TMS)	0.218 ± 0.003	0.479 ± 0.024	0.72 ± 0.019	0.999
36	Proline (2TMS)	0.261 ± 0.009	0.525 ± 0.026	0.703 ± 0.037	0.987
38	Pyruvic acid (1metho-oxim, 1TMS)	0.013 ± 0.003	0.029 ± 0.007	0.048 ± 0.012	0.995
40	Serine (3TMS)	0.068 ± 0.005	0.13 ± 0.004	0.167 ± 0.007	0.978
42	Succinic acid (2TMS)	0.02 ± 0	0.025 ± 0.001	0.025 ± 0.001	0.771
44	Threonine (3TMS)	0.054 ± 0.004	0.111 ± 0.006	0.147 ± 0.009	0.985
47	Tryptophan (2TMS)	0.011 ± 0.002	0.02 ± 0.001	0.023 ± 0.003	0.903
48	Tyrosine (3TMS)	0.087 ± 0.009	0.194 ± 0.012	0.287 ± 0.025	0.999
50	Valine (2TMS)	0.256 ± 0.021	0.5 ± 0.039	0.706 ± 0.059	0.998
